# A comparative cost analysis of TIPS and BRTO for secondary prophylaxis in gastric variceal bleeding

**DOI:** 10.1186/s42155-025-00625-z

**Published:** 2025-12-10

**Authors:** Warren Clements, Abigail Chenoweth, Salam Findakly, Tuan D. Phan, Mark Bolger, William P. L. Bradley, William Kemp, Stuart K. Roberts, Matthew W. Lukies, Gerard S. Goh, Tim Joseph, Christine Ball, Jim Koukounaras

**Affiliations:** 1 Department of Radiology, Alfred Hospital, Melbourne, VIC 3004 Australia; 2 Department of Surgery, School of Translational Medicine, Monash University, Melbourne, VIC 3004 Australia; 3 National Trauma Research Institute, Alfred Health, Melbourne, Victoria 3004 Australia; 4 Department of Anaesthesiology and Perioperative Medicine, Alfred Health, Melbourne, VIC 3004 Australia; 5 Department of Anaesthesiology and Perioperative Medicine, School of Translational Medicine, Faculty of Medicine, Nursing and Health Sciences, Monash University, Melbourne, VIC 3004 Australia; 6 Department of Gastroenterology, Alfred Hospital, Melbourne, VIC 3004 Australia; 7 Department of Gastroenterology, School of Translational Medicine, Monash University, Melbourne, VIC 3004 Australia; 8 Department of Medical Imaging, Monash Health, Melbourne, VIC 3168 Australia

**Keywords:** TIPS, BRTO, Varices, Embolization, Cost

## Abstract

**Background:**

After a gastric variceal rupture, clinical practice guidelines recommend either transjugular intrahepatic portosystemic shunt (TIPS) or balloon-occluded retrograde transvenous obliteration (BRTO) as interventional options for secondary prevention. This study aimed to generate and compare TIPS and BRTO costing data to calculate the secondary prevention cost following gastric variceal bleeding, to encourage consideration of resource cost within decision-making.

**Methods:**

All costs were included for patients treated between 1 January 2017 and 1 January 2024. Data on procedure, non-procedure, and ward costs were collected. Ward costs were only calculated for elective admissions to reduce bias from inpatient emergency procedures. All costs were measured from a healthcare system perspective and included direct and indirect expenses where relevant. A 5% indexation for inflation was applied to non-fixed costs from 2017 to the 2024 cost year.

The cost to prevent gastric re-bleeding was then calculated by adjusting the costing data generated based on existing outcome data in the literature, utilising the largest existing meta-analysis on the efficacy of TIPS and BRTO in preventing re-bleeding.

**Results:**

There were 38 patients in the study cohort, with a mean age 56.8 years (*SD* 12.0), and 25 patients (66%) were male. TIPS was performed in 27 patients (71%). The TIPS and BRTO groups had similar mean age, proportion of male sex, CP grading, and proportions of elective admissions.

The median total cost for TIPS was AUD$11,922 (range $6307–$53,432), while the median total cost for BRTO was AUD$3632 (range $1818–$5174), *p* < 0.001. The adjusted cost to prevent future gastric re-bleed using TIPS was AUD$14,803, while the cost to prevent re-bleed using BRTO was AUD$3896.

**Conclusion:**

The cost magnitude of both TIPS and BRTO was both low in an Australian model, and both remain good options for patients. However, the use of BRTO was associated with significantly lower upfront procedural costs than for TIPS for secondary prevention of gastric variceal bleeding. Costs should form a key component of the value of IR to modern healthcare.

## Introduction

Gastric varices are common in the setting of portal hypertension, owing to the development of natural porto-systemic shunts (PSS) aiming to decompress portal pressure by diverting splanchnic blood to the systemic venous circulation [1]. While less common than oesophageal varices, gastric varices are prone to rupture at a lower pressure threshold, and mortality post-rupture can be as high as 60% [2].

In Australia, portal hypertension is most commonly the result of advanced chronic liver disease with cirrhosis [3, 4]. Once a patient decompensates from clinically significant portal hypertension (CSPH, defined as a hepatic venous pressure gradient (HVPG) at or above 10 mmHg) [5], their median survival decreases to less than 18 months [4].

As detailed in the American Association for the Study of Liver Disease (AASLD) and the European Association for the Study of the Liver (EASL) Clinical Practice Guidelines, acute variceal haemorrhage is an emergency, and 6-week mortality can approach 15% [6, 7]. Initial management includes endoscopy, vasoactive therapy, and intravenous antibiotics [6]. Endoscopic treatment for oesophageal varices has a high success rate, while conversely it is a greater challenge for patients with gastric varices owing to several systemic shunts and faster porto-systemic blood flow [3]. In patients where endoscopic treatment fails, or where it is successful but the patient remains high risk (*HVPG* > 20 mmHg, Child–Pugh (CP) C, or CP B with active bleeding on endoscopy), there is a role for acute transjugular intrahepatic portosystemic shunt (TIPS), which works to decompress the existing PSS and has been shown to improve long-term mortality through reduction of future bleeding events [8, 9]. In this setting, TIPS may be combined with antegrade embolisation [8]. However, TIPS poses its own challenges with itself carrying a risk of procedural complication of approximately 5%, procedural mortality of approximately 1%, and a risk of encephalopathy of up to 50% [1]. It also requires significant staff and capital resources, which potentially increases costs.

Newer interventional radiology (IR) techniques have been developed targeting specific variceal embolisation. These may be performed retrograde without the need for transhepatic access and can be performed using a selection of cyanoacrylate, foam sclerosant, balloon-occlusion catheters, plugs, or coils [10]. The most commonly known technique is called balloon-occluded retrograde transvenous obliteration (BRTO), which is specifically performed using a balloon-occlusion catheter via a transrenal shunt [11]. These embolisation techniques offer a more direct target of the culprit varix but do not address the specific underlying portal hypertension. While they are generally less invasive and have a lower procedural mortality than TIPS, they may also increase portal pressure, leading to precipitation of ascites or worsening of other PSS [11].

The relative cost of BRTO has not been established in an Australian healthcare setting. Costs form an important component of the relative value of minimally invasive techniques that IR offers, and it is crucial that the relative costs of our treatments are reported so as to divert resources, funding, and capital to cost-effective services [12].

After an initial gastric variceal bleeding episode, secondary prophylaxis techniques should be adopted to reduce the risk of re-bleeding and short-to-intermediate term mortality. This may include nonselective beta blockers, endoscopic surveillance, banding of high-risk oesophageal varices, and targeted therapy to gastric varices. EASL and AASLD both recommend using TIPS or RTO according to individual patient clinical and anatomic factors [7, 8]. A recent systematic review and meta-analysis from Wang et al. assessed and compared the use of TIPS and BRTO in the setting of gastric varices [13]. In assessing nine studies including eight randomised and controlled trials, the authors showed a higher overall survival when using BRTO (*RR* 0.81, 95% *CI* 0.66–0.98, *p* = 0.03) and a higher rebleeding rate in the TIPS group (*RR* 2.61, 95% *CI* 1.75 to 3.9, *p* < 0.00001). The authors showed no difference in the rate of immediate haemostasis, technical success, aggravated ascites, or change to the Child–Pugh stage.

This study aimed to generate and compare costing data on TIPS and BRTO in an Australian healthcare model, to calculate and compare the upfront index procedure cost of the use of these techniques for secondary prevention of gastric variceal bleeding using existing level 1 efficacy data. It is intended that this data will encourage consideration of resource cost within decision making.

## Materials and methods

### Ethics

The Alfred Hospital Research and Ethics Committee approved this costing study, which included a waiver of patient consent, approval number 511/24. This retrospective study conforms to the STROBE checklist.

### Data collection

Patients were identified through the radiology information system (RIS), and procedure data were collated from RIS and the electronic medical record (EMR). A combination of internal ordering systems (Prospitalia H-track, Richmond, Australia), the Victorian Cost Data Collection (VCDC), and the Victorian Admitted Episodes Dataset (VAED) were used to collect cost data. Individual re-bleed outcome data was not collected, given the study type and the aims of the study.

### Inclusion and exclusion criteria

Patients treated using TIPS or BRTO between the 7 years of 1 January 2017 and 1 January 2024 were suitable for inclusion, regardless of the indication for the procedure, given the study assessed only upfront costs and not procedural efficacy. Where antegrade variceal embolisation techniques were employed, patients were excluded.

Data on procedure costs were collated from all included patients. Ward costs included parameters such as overheads, nursing, food, allied health, and support costs. Non-procedure costs included peri-procedural adjuncts such as blood testing, blood product transfusion, and pharmacy costs. Data on ward costs and non-procedure costs were only included for elective admissions; this was done to reduce bias from procedures done in an acute setting during a more prolonged inpatient admission, where the long admission costs might not be a true reflection of the up-front costing. For example, ward costs were not included when TIPS or BRTO were performed as secondary prevention at the end of a long inpatient admission. The authors acknowledge this may underestimate costs for emergency TIPS, although it is more likely to provide a more real-world costing figure.

### Treatment approach

In our centre, TIPS is performed using a standard fluoroscopic-guided approach, as described [14] using the Rösch-Uchida Transjugular Liver Access Set (Cook Medical, Bloomington, IN, USA) and the Viatorr TIPS endoprosthesis (WL Gore, Newark, DE, USA). Where difficult anatomy was encountered, or the procedure was initially unsuccessful in accessing the portal vein, a gunsight approach was used as published [15]. For BRTO procedures, retrograde occlusion was obtained using a balloon-occlusion catheter (Python 7Fr, LeMaitre Medical, Burlington, MA, USA), and embolisation was performed using foam sclerotherapy with 3% Aethoxysklerol made to a foam using a 1:1 dilution with air (Polidocanol, Getz Healthcare, Lane Cove, NSW, Australia). A 30-min balloon inflation time was used as previously published [2].

### Costing

All costs shown are from a healthcare system perspective and expressed in Australian dollars (AUD$). As described in previous publications [16, 17], procedure costing was generated by assessing bottom-up costs using a record of equipment from the Prospitalia H-track database. This included procedure time and staffing costs with 5% indexation for inflation using costs generated in 2017 up to the 2024 price year [18]. For patients treated with IR-led conscious sedation, this is calculated as procedure time (hours) multiplied by $502.70 plus indexation. For anaesthetic cases, this was calculated as procedure time (hours) multiplied by $752.88 plus indexation, with all wages based on existing enterprise bargaining agreements. All other costs were generated top-down using the VCDC and VAED datasets, including staff wages, ICU costs, ward-based care, pharmacy, blood products, and pathology. These were separated into ward costs and all other non-procedure costs.

### Cost-outcome analysis

The cost to prevent one gastric re-bleed was calculated using the costing data generated in this study, based on outcome data from the largest meta-analysis on the efficacy of TIPS and BRTO in preventing re-bleeding. Based on the study of Wang et al., the rate of variceal-specific re-bleeding following TIPS was 19.5%, and the rate following BRTO was 6.9% [13].

### Statistical analysis

Costing data were collated into Microsoft Excel (Microsoft, USA) and analysed using Stata (Version 18.0-BE, StataCorp, TX, USA). As appropriate to the type, data were presented using mean and standard deviation (SD), median and range, or frequency and percentage. Statistical comparison between the TIPS and BRTO groups was performed using Student’s *t*-test, Mann–Whitney *U*-test, or chi-square test with a two-tailed probability value of less than 0.05 deemed significant.

## Results

There were 38 patients in the study cohort, with a mean age of 56.8 years (*SD* 12.0), and 25 patients (65.8%) were of male sex. Most patients had Child–Pugh B cirrhosis (19, 50.0%), and the most common cause of cirrhosis was alcohol overuse (23, 60.5%). TIPS was used more commonly (27 patients, 71.1%), and the median length of stay was 5 days (range 0–48). Further summary statistics are shown in Table 1.

**Table 1:** Summary statistics of the whole cohort

Variable	Value
Total number of patients	38
Age in years (mean, SD)	56.8 (12.0)
Male sex (number, percentage)	25 (65.8%)
Procedure time in minutes (median, range)	145 (65–365)
Child–Pugh grade (number, percentage)	No cirrhosis: 1 (2.6%) A: 7 (18.4%) B: 19 (50.0%) C: 11 (29.0%)
Model for End-Stage Liver Disease (MELD) score, version 3.0 (median, range)	15 (8–26)
Reason for liver disease (number, percentage)	Hepatitis C: 3 (7.9%) Hepatitis B: 0 (0%) Alcohol: 23 (60.5%) MAFLD^a^: 3 (7.9%) Haemochromatosis: 0 (0%) More than one cause: 6 (15.8%)
Type of procedure performed (number, percentage)	TIPS^b^: 27 (71.1%) BRTO^c^ : 11 (28.9%)
Elective procedures (number, percentage)	16 (42.1%)

^a^*MAFLD*, metabolic dysfunction-associated fatty liver disease

^b^*TIPS* transjugular intrahepatic portosystemic shunt

^c^*BRTO* balloon-occluded retrograde transvenous obliteration

In comparing the unmatched groups as shown in Table 2, the TIPS and BRTO groups had similar mean age, proportion of male sex, stage of cirrhosis, Model for End-Stage Liver Disease (MELD) score, and proportion of elective admissions. However, the TIPS group was associated with a longer median procedure time (185 vs 120 min, *p* = 0.004), a longer median length of hospital stay (2 vs 0 days, *p* = 0.001), and a higher use of anaesthesiology (100% vs 18.2%, *p* < 0.001). The TIPS group also had a higher proportion of periprocedural complications (22.2% vs 0%, *p* = 0.088) and a higher all-cause 30-day mortality (18.5% vs 0%, *p* = 0.126).

**Table 2:** Summary of treatments comparing transjugular intrahepatic portosystemic shunt (TIPS) and balloon-occluded retrograde transvenous obliteration (BRTO)

Variable	TIPS	BRTO	*p*-value
Total treatments	27	11	N/A
Age in years (mean, SD)	54.7 (11.6)	62.0 (11.7)	0.090
Male sex (number, percentage)	17 (68.0%)	10 (76.9%)	0.565
Child–Pugh status (number, percentage)	No cirrhosis: 0 (0%) A: 3 (11%) B: 14 (52%) C: 10 (37%)	No cirrhosis: 1 (9%) A: 4 (36%) B: 5 (45%) C: 1 (9%)	0.062
Model for End-Stage Liver Disease (MELD) score, version 3.0 (median, range)	15 (8–26)	15 (8–22)	0.205
Procedure time in minutes (median, range)	185 (105–365)	120 (65–185)	0.004
Nontraditional TIPS technique^a^ required (number, percentage)	7 (25.9%)	N/A	N/A
Elective admission (number, percentage)	10 (37.0%)	6 (54.5%)	0.321
Length of hospital stay in nights^b^ (median, range)	2 (1–14)	0 (0–1)	0.001
Procedural complication (number, percentage)	1 (3.7%)	0 (0%)	0.518
Peri-procedural complication (number, percentage)	6 (22.2%)	0 (0%)	0.088
Required anaesthetics (number, percentage)	27 (100%)	2 (18.2%)	< 0.001
All-cause 30-day mortality (number, percentage)	5 (18.5%)	0 (0%)	0.126
Procedure costs per patient in $AUD (median, range)	9577 (4545–15,525)	2879 (1381–3899)	< 0.001
Non-procedure costs^b^ per patient in $AUD (median, range)	1497 (869–11,305)	378 (127–733)	< 0.001
Ward costs^b^ per patient in $AUD (median, range)	2074 (981–26,689)	410 (309–812)	< 0.001
**Total cost per treatment in $AUD (median, range)**	**11,922 (6307**–**53,432)**	**3632 (1818**–**5174)**	** < 0.001**

^a^Gunsight TIPS approach when traditional TIPS is unsuccessful [14]

^b^Calculated for elective admissions only

Further clarifying the six complications in the TIPS group, one patient suffered intraperitoneal haemorrhage requiring ICU admission following the procedure which resulted in mortality during the admission (CIRSE grade 6), three patients suffered from ongoing variceal haemorrhage requiring further variceal-specific embolisation (CIRSE grade 3), one patient suffered from encephalopathy (CIRSE grade 4), and one patient suffered from acute TIPS thrombosis requiring revision (CIRSE grade 3).

**Fig. 1:**
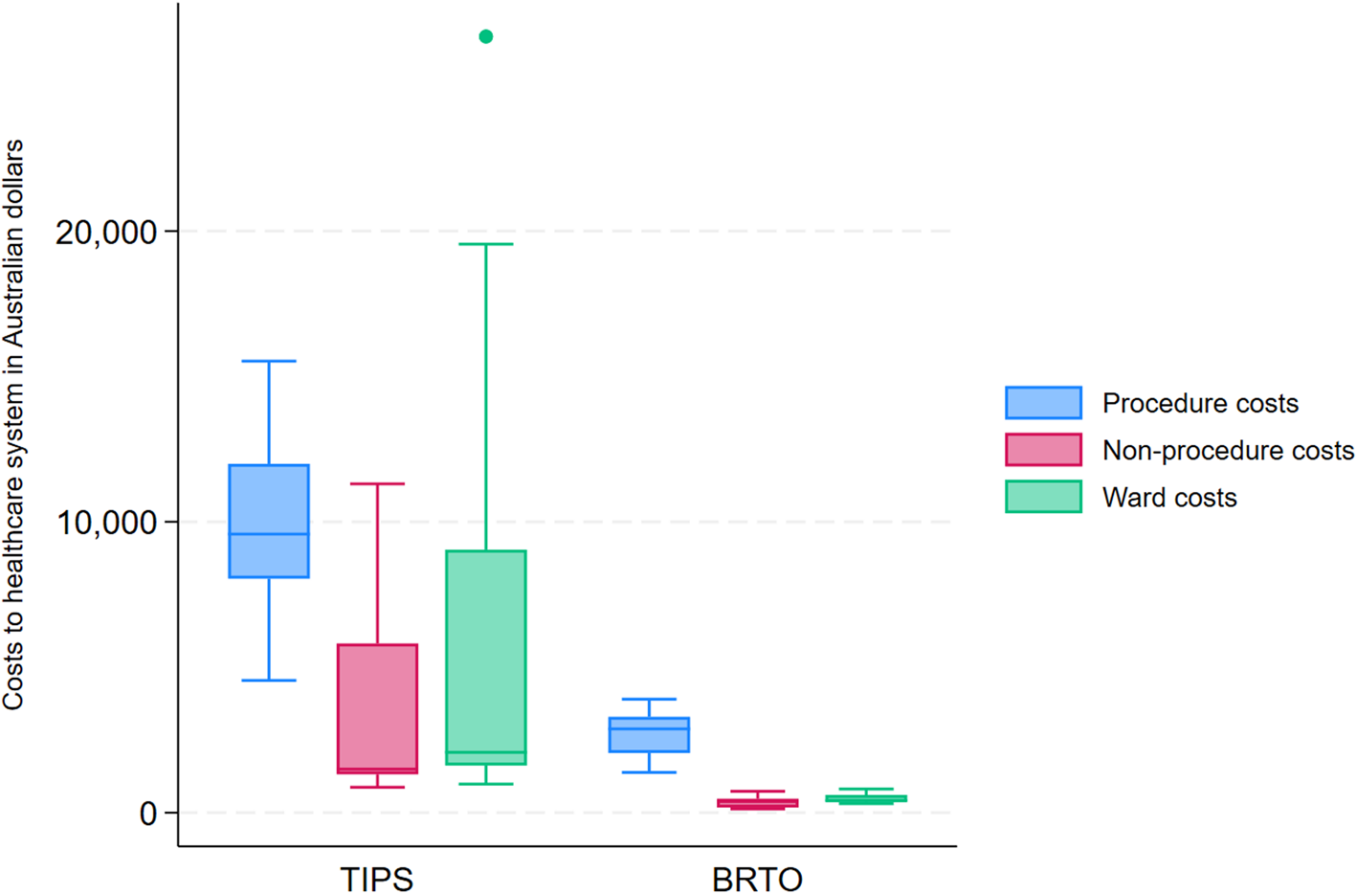
Box and whisker plots comparing the individual component costs of the transjugular intrahepatic portosystemic shunt (TIPS) and balloon-occluded retrograde transvenous obliteration (BRTO) groups

In assessing the primary endpoint, the TIPS group was associated with higher median procedure costs (AUD$9577 vs AUD$2879, *p* < 0.001), higher median non-procedure costs (AUD$1497 vs AUD$378, *p* < 0.001), and higher ward-based costs (AUD$2074 vs AUD$410, *p* < 0.001). These are shown in Fig. 1. The overall median total cost for TIPS was AUD$11,922 (range 6307–53,432), while the median total cost for BRTO was AUD$3632 (range 1818–5147), *p* < 0.001.

Using variceal-specific rebleed data from the study of Wang et al. [13], the cost to prevent one re-bleed using TIPS was a median of AUD$14,803, while the cost to prevent one re-bleed using BRTO was AUD$3896. The incremental cost-outcome is not shown, as BRTO was both lower cost and higher survival, resulting in an unequivocally positive costing outcome.

### Matched subanalysis

A sub-analysis was also performed on 10 age, sex, and Child–Pugh stage-matched patients, with results shown in Table 3. The cost differences were similar to the unmatched cohort (TIPS AUD$17,634 vs BRTO AUD$3988, *p* = 0.009).

**Table 3:** Summary of age, sex, and Child–Pugh matched elective patients comparing transjugular intrahepatic portosystemic shunt (TIPS) and balloon-occluded retrograde transvenous obliteration (BRTO)

Variable	TIPS	BRTO	*p*-value
Total treatments	5	5	N/A
Age in years (mean, SD)	65.4 (7.9)	68.2 (4.1)	0.493
Male sex (number, percentage)	4 (80%)	4 (80%)	1.000
Child–Pugh status (number, percentage)	A: 2 (40%) B: 3 (60%) C: 0 (0%) No cirrhosis: 0 (0%)	A: 3 (60%) B: 2 (40%) C: 0 (0%) No cirrhosis: 0 (0%)	0.527
Modified End-Stage Liver Disease (MELD) score, version 3.0 (median, range)	10 (9–20)	15 (8–19)	0.916
Procedure time in minutes (median, range)	210 (125–365)	100 (65–185)	0.075
Nontraditional TIPS technique^a^ required (number, percentage)	2 (40%)	N/A	N/A
Length of hospital stay in nights^b^ (median, range)	2 (1–14)	0 (0)	0.005
Procedural complication (number, percentage)	1 (20%)	0 (0%)	0.292
Peri-procedural complication (number, percentage)	2 (40%)	0 (0%)	0.114
Procedure costs per patient in $AUD (median, range)	8332 (7422–15,437)	2844 (1382–3629)	0.009
Non-procedure costs^b^ per patient in $AUD (median, range)	1561 (870–11,306)	331 (127–476)	0.009
Ward costs^b^ per patient in $AUD (median, range)	2194 (1327–26,689)	397 (310–812)	0.009
**Total cost per treatment in $AUD (median, range)**	**17,634 (11,548**–**51,211)**	**3988 (1931**–**4365)**	**0.009**

^a^Gunsight TIPS approach when traditional TIPS is unsuccessful [14]

^b^Calculated for elective admissions only

## Discussion

This study showed that the magnitude of both TIPS and BRTO as a cost-outcome analysis were both low in an Australian model, and both remain good options for patients. However, the use of BRTO was associated with significantly lower upfront procedural costs than for TIPS, and this is noting that the groups were both similar in terms of background demographics, confirming this with a cohort matched for age, sex, and Child–Pugh status.

In comparing the magnitude of these costs with existing studies, the cost is far lower than many studies from the USA, with reported costs ranging from approximately US $21,000 to US $150,000 depending on the methods used to assess costs and the individual healthcare systems [19–22]. The costs for TIPS in this study are slightly higher than a recent UK study where, in the setting of recurrent ascites, the authors quote a median TIPS cost of £2564.58; however, it is not clear whether the study of Parker et al. included peri-procedural costs, ward costs, and prosthesis costs, particularly given their median cost is lower than the cost of the endoprosthesis alone at AUD$4000 [23]. The costs in our study are somewhat comparable to a 2023 Spanish study where the authors suggest a cost of TIPS for variceal bleeding of 7527.79 € [24].

To date, limited studies have assessed the cost of BRTO and related retrograde embolisation procedures, possibly due to its lack of popularity in the Western Hemisphere. A study from Japan in 2017 evaluated the cost of equipment used to perform BRTO, quoting figures between ¥83,120 and ¥205,024, similar to those in our study; however, the authors use liquid 5% ethanolamine oleate and a 4-h balloon-inflation time, which would likely result in slightly higher ancillary and peri-procedural costs, which were not measured [25]. There are currently no costing studies in an Australian healthcare model.

The authors believe that the costs presented in this study will be translatable to the broader IR community including outside Asia and Australia, despite their local currency value. This is because costs and cost differentials will be proportional even if the magnitude of costs is different in different healthcare settings. We believe this is also benefited by using a resource-driven costing model rather than a payer model, with the latter very heavily influenced by costs based on the individual healthcare system. In this context, IRs worldwide should be able to incorporate this data into their clinical decision-making.

Additional relative costs in the TIPS group are likely accountable by the longer procedure time, more frequent use of anaesthesiology, longer ward stay, and the very high cost of the endoprosthesis. However, the TIPS group has a higher proportion of Child-Pugh C patients, which may also be a factor. In fact, the TIPS endoprosthesis itself incurred a higher cost than the median total cost for the entire BRTO procedure, including all ancillary BRTO costs. While TIPS may seem inferior in this setting, we must also consider this in the context of the inherent differences between how the two procedures achieve their goal and their effect on underlying portal pressures [26]. TIPS can treat not just gastric varices but portal hypertension and its associated symptoms, such as ascites. Given that TIPS decompresses the entire portal system and can concurrently improve symptoms such as ascites, it may be preferable in patients with extensive disease or refractory ascites. Conversely, BRTO may be more suitable for patients with isolated gastric varices and relative contraindications to TIPS (e.g. high risk of encephalopathy) given its targeted approach and potentially lower overall complication risk. In addition, BRTO requires suitable anatomy, so it may not be appropriate for all patients. TIPS also requires suitable anatomy but is less prone to selection biases, and with the advancement of techniques such as gunsight, it can be offered to more patients than in the past [15]. While BRTO does not treat the underlying portal hypertension, its ability to specifically target the site of precipitant bleeding is shown in the level 1 data on its efficacy, and thus, combining BRTO with active medical management is a low-cost and reasonable approach for patients. As a newer concept, BRTO may be still gaining traction in clinical practice.

The BRTO group showed a lower use than TIPS in our cohort, despite EASL and the AASLD recommending both as suitable secondary prevention techniques [7, 8]. There may be several factors to consider in this context, including hospitals not offering the procedure, different perceptions of the technique, and lack of multidisciplinary input [26]. Patients with variceal bleeding should be discussed in a multidisciplinary setting, which includes the expertise of interventional radiologists, interventional endoscopists, hepatologists, and anaesthetists. There may be a role in combining TIPS with BRTO (or other embolisation/endoscopic techniques), which combines advantages from both procedures; however, conversely, this still exposes some TIPS-related risks (e.g. encephalopathy) plus incurs TIPS-related costs, including the endoprosthesis. As more data evolves on the combined use of these techniques, improvements in long-term mortality could justify potentially increased costs.

Given TIPS has been performed for a long time, there is existing patient-reported outcome data, including quality-adjusted life years (QALYs). Unfortunately, this data is unavailable for BRTO, which prohibited a specific cost-effectiveness analysis and why this study reports cost-outcome as its primary endpoint. It is crucial that QALY data is generated moving forward, specifically in an Australian setting, given the patient population with chronic liver disease and underlying demographics do vary across the globe according to local lifestyle and genetic influences.

The study notes several limitations, including the imbalanced case mix and urgency, the use of elective-only costing for ward costs, variability of costs in different healthcare models, and the lack of inclusion of longer-term indirect costs, including costs to manage complications. This does offset the relatively high complication rate in the TIPS cohort, which did not contribute to costs directly in the study given its inherent design. The study is single-site and used existing published data on clinical treatment success rather than our own outcome data. However, this was done to improve the reliability of the endpoints, as our population was too small to provide a reliable and efficacy endpoint. In this context, it is standard for a costing study to use external efficacy data.

In conclusion, this study fills an important gap in the literature by providing up-to-date, real-world cost data for BRTO within the Australian healthcare system—an area that has been largely unexamined. This includes the relatively lower cost when using BRTO as secondary prevention of gastric bleeding compared to TIPS. These findings offer clinicians and healthcare administrators critical evidence on resource allocation and cost-effectiveness, helping guide decisions on whether to adopt BRTO as a lower-cost alternative to TIPS for secondary prophylaxis in gastric variceal bleeding.

## Data Availability

The datasets generated and/or analysed during the current study are not publicly available as they were not provided by the ethical committee consent process, but are available from the corresponding author on reasonable request.

## References

[1] Liang A, Brar S, Almaghrabi M, Khan MQ, Qumosani K, Teriaky A. Medicine. 2023;102(38):e35266. 37746955 10.1097/MD.0000000000035266 PMC10519530

[2] Clements W, Barrett R, Roberts SK, Majeed A, Kemp W, Moriarty HK. Balloon‐occluded retrograde transvenous obliteration (BRTO) of gastric varices using foam sclerosant and a reduced balloon inflation time: feasibility and efficacy. J Med Imaging Radiat Oncol. 2020;64(4):490–5. 32441461 10.1111/1754-9485.13049

[3] Clements W, Cavanagh K, Ali F, Kavnoudias H, Kemp W, Roberts S, et al. Variant treatment for gastric varices with polidocanol foam using balloon‐occluded retrograde transvenous obliteration: a pilot study. J Med Imaging Radiat Oncol. 2012;56(6):599–605. 23210578 10.1111/j.1754-9485.2012.02453.x

[4] D’Amico G, Garcia-Tsao G, Pagliaro L. J Hepatol. 2006;44(1):217–31. 16298014 10.1016/j.jhep.2005.10.013

[5] Turco L, Villanueva C, La Mura V, García-Pagán JC, Reiberger T, Genescà J, et al. Clin Gastroenterol Hepatol. 2020;18(1):313–27. 31176013 10.1016/j.cgh.2019.05.050

[6] Abraldes JG, Berzigotti A, Bosch J. Portal hypertensive bleeding in cirrhosis: risk stratification, diagnosis, and management: 2016 practice guidance by the American Association for the Study of Liver Diseases. Hepatology. 2017;65(1):310–35. 27786365 10.1002/hep.28906

[7] Bureau C, Larrue H, Cortes-Cerisuleo M, Miraglia R, Procopet B, Rudler M, Trebicka J, VanWagner LB, Hernandez-Gea V. EASL clinical practice guidelines on TIPS. J hepatol. 2025;83(1):177–210. 10.1016/j.jhep.2025.01.029 40180845

[8] Lee EW, Eghtesad B, Garcia-Tsao G, Haskal ZJ, Hernandez-Gea V, Jalaeian H, et al. Hepatology. 2024;79(1):224–50. 37390489 10.1097/HEP.0000000000000530

[9] Kaplan DE, Ripoll C, Thiele M, Fortune BE, Simonetto DA, Garcia-Tsao G, et al. Hepatology. 2024;79(1):1180–211. 37870298 10.1097/HEP.0000000000000647

[10] Saad WE, Sabri SS. Balloon-occluded retrograde transvenous obliteration (BRTO): technical results and outcomes. Semin Intervent Radiol. 2011;28(03):333–8. 22942551 10.1055/s-0031-1284460 PMC3312162

[11] Kim YH, Kim YH, Kim CS, Kang UR, Kim SH, Kim JH. Cardiovasc Intervent Radiol. 2016;39:840–6. 26757912 10.1007/s00270-015-1288-8

[12] Clements W. Cardiovasc Intervent Radiol. 2024;47(2):158–60. 38147154 10.1007/s00270-023-03637-1

[13] Wang ZW, Liu JC, Zhao F, Zhang WG, Duan XH, Chen PF, et al. Comparison of the effects of TIPS versus BRTO on bleeding gastric varices: a meta‐analysis. Can J Gastroenterol Hepatol. 2020;2020(1):5143013. 32104670 10.1155/2020/5143013 PMC7036113

[14] Gaba RC, Khiatani VL, Knuttinen MG, Omene BO, Carrillo TC, Bui JT, et al. Comprehensive review of TIPS technical complications and how to avoid them. AJR Am J Roentgenol. 2011;196(3):675–85. 21343513 10.2214/AJR.10.4819

[15] Lukies M, Moriarty H, Phan T. Modified gun-sight transjugular intrahepatic portosystemic shunt technique. Br J Radiol. 2022;95(1140):20220556. 36063126 10.1259/bjr.20220556 PMC9733616

[16] Clements W, Chenoweth A, Morphett L, Billington E, Nandurkar R, Phan T, et al. A cost outcome study of varicocoele embolisation and future pregnancy in an Australian public hospital setting. J Med Imaging Radiat Oncol. 2024;68(3):282–8. 38437182 10.1111/1754-9485.13629

[17] Clements W, Chenoweth A, Phipps B, Mozo L, Bolger M, Morphett L, et al. A study comparing the cost‐effectiveness of conventional and drug‐eluting transarterial chemoembolisation (c TACE and DEB‐TACE) for the treatment of hepatocellular carcinoma in an Australian public hospital. J Med Imaging Radiat Oncol. 2024;68(6):714–20. 38985987 10.1111/1754-9485.13731

[18] Clements W, Moriarty HK, Koukounaras J, Joseph T, Phan T, Goh GS. The cost to perform uterine fibroid embolisation in the Australian public hospital system. J Med Imaging Radiat Oncol. 2020;64(1):18–22. 10.1111/1754-9485.12982. 31793208 10.1111/1754-9485.12982

[19] Russo MW, Zacks SL, Sandler RS, Brown JRS. Cost-effectiveness analysis of transjugular intrahepatic portosystemic shunt (TIPS) versus endoscopic therapy for the prevention of recurrent esophageal variceal bleeding. Hepatology. 2000;31(2):358–63. 10655258 10.1002/hep.510310215

[20] Shen NT, Schneider Y, Congly SE, Rosenblatt RE, Namn Y, Fortune BE, et al. Cost effectiveness of early insertion of transjugular intrahepatic portosystemic shunts for recurrent ascites. Clin Gastroenterol Hepatol. 2018;16(9):1503–10. 29609068 10.1016/j.cgh.2018.03.027

[21] Boyer TD, Henderson JM, Heerey AM, Arrigain S, Konig V, Connor J, Abu-Elmagd K, Galloway J, Rikkers LF, Jeffers L, DIVERT Study Group. Cost of preventing variceal rebleeding with transjugular intrahepatic portal systemic shunt and distal splenorenal shunt. J hepatol. 2008;48(3):407–14. 10.1016/j.jhep.2007.08.014 PMC2743029 18045724

[22] Kuei A, Lee EW, Saab S, Busuttil RW, Durazo F, Han SH, et al. Inpatient cost assessment of transjugular intrahepatic portosystemic shunt in the USA from 2001 to 2012. Dig Dis Sci. 2016;61(10):2838–46. 10.1007/s10620-016-4233-z. 27349987 10.1007/s10620-016-4233-z

[23] Parker MJ, Guha N, Stedman B, Hacking N, Wright M. Trans-jugular intrahepatic porto-systemic shunt placement for refractory ascites: a ‘real-world’ UK health economic evaluation. Frontline Gastroenterol. 2013;4(3):182–6. 28839725 10.1136/flgastro-2012-100283 PMC5370052

[24] Bañares R, Albillos A, Nakum M, Gea S, Varghese A, Green W. An economic analysis of transjugular intrahepatic portosystemic covered stent shunt for variceal bleeding and refractory ascites in a Spanish setting. Adv Ther. 2023;40(7):3006–20. 10.1007/s12325-023-02517-x. 37160834 10.1007/s12325-023-02517-x PMC10272260

[25] Takenaga S, Ashida H, Matsui Y, Fukuda K. Balloon-occluded retrograde transvenous obliteration for gastric varices: efficacy of coaxial double-balloon catheter system. Jpn J Diagn Imaging. 2017;35(2):118–24.

[26] Kalo E, Read S, George J, Roberts SK, Majumdar A, Ahlenstiel G. Attitudes towards transjugular intrahepatic portosystemic shunt (TIPS) in Australia: a national survey of TIPS centres. BMJ Open Gastroenterol. 2024;11(1):e001308. 38519047 10.1136/bmjgast-2023-001308 PMC10966807

